# A Patient of Multiple Myeloma with Absent M-spike on Serum Protein Electrophoresis and Elevated Serum-Free Light Chains: A Case Report and Literature Review

**DOI:** 10.7759/cureus.5398

**Published:** 2019-08-16

**Authors:** Areej Lalani, Kashif Aziz, Mehreen Khan, Tayyaba Zubair, Syed Ijlal Ahmed

**Affiliations:** 1 Internal Medicine, Dow Medical College and Civil Hospital Karachi, Dow University of Health Sciences, Karachi, PAK; 2 Neurology, Jersey Neurosciences, New Jersey, USA; 3 Internal Medicine, George Washington University School of Medicine and Health Sciences, Washington DC, USA; 4 Internal Medicine, Desai Medical Center, Ellicott City, USA; 5 Neurology, Liaquat National Hospital and Medical College, Karachi, PAK

**Keywords:** multiple myeloma, serum protein electrophoresis, free light chains, immunofixation, plasma cells, bone pain, pathologic fractures, erythrocyte sedimentation rate

## Abstract

Multiple myeloma is a neoplasm described as an abnormal growth of plasma cells that outnumbers the other normal hematopoietic cells inside the bone marrow. Patients are diagnosed at a median age of 66-77 years with 37% of those with age less than 65. Unexplained bone pain (most commonly in back and ribs), pathologic fractures, fatigue, and weight loss are common initial symptoms at presentation. Here, we report a rare presentation of multiple myeloma with normal serum protein electrophoresis but elevated serum-free light chains. The absence of monoclonal gammopathy on protein electrophoresis or normal immunofixation does not negate the diagnosis of multiple myeloma. Therefore, all the sub types of multiple myeloma need to be comprehensively studied to aid in reaching an accurate diagnosis.

## Introduction

Multiple myeloma is a neoplasm described as an abnormal growth of plasma cells that outnumbers the other normal hematopoietic cells inside the bone marrow. These clonal plasma cells synthesize and secrete unusually large quantities of abnormal immunoglobulin that can result in end-organ dysfunction [1]. Patients are diagnosed at a median age of 66-77 years with 37% of those with age less than 65 [2]. Unexplained bone pain (most commonly in back and ribs), pathologic fractures, fatigue, and weight loss are common initial symptoms at presentation. Some patients may only present with abnormal laboratory tests like anemia, hypercalcemia, or increased protein levels. Diagnostic workup will include differential complete blood count (CBC), beta-2 microglobulin tests, immunoglobulin studies, skeletal survey, and bone marrow biopsy [3]. The treatment plan consists of oncology referral for chemotherapy and bone marrow stem cell transplant consideration. Here, we report a rare presentation of symptomatic multiple myeloma with normal serum protein electrophoresis (SPEP) but elevated serum-free light chains during serum immunofixation.

## Case presentation

A 55-year-old male, referred to our clinic with a complaint of a three-year history of progressive lumbar back pain, worsening in intensity since the past few months. The patient was responding poorly to multiple strong analgesic medications. Upon further questioning, the patient also revealed an unintentional weight loss of seven to ten kilograms over two years. The patient had no other comorbidities and was in a good state of health otherwise. On physical examination, there was an absence of tenderness over the spine. Rest of the physical examination was also overall unremarkable. Basic laboratory investigations including CBC, serum electrolytes, erythrocyte sedimentation rate (ESR), and renal function tests were ordered. The results are summarized in Table 1.

**Table 1:** Basic laboratory investigations RBC: Red blood cell; MCV: Mean corpuscular volume; MCH: Mean corpuscular hemoglobin; ESR: Erythrocyte sedimentation rate; WBC: White blood cell

TEST	RESULT	REFERENCE RANGE
Hemoglobin	10.7 g/dl	13-17
RBC count	3.3 million/cmm	4.5-5.5
Hematocrit	33%	40-50
MCV	99 fL	83-101
MCH	32 pg.	27-32
Total WBC count	5620 cells/mm3	4000-10500
ESR	101 mm/hr	0-10
Serum creatinine	0.70 mg/dl	0.5-1.2
Sodium	139 mmol/L	135-148
Potassium	4.1 mm/L	3.5-5
Chloride	99 mm/L	98-106

He had normochromic normocytic anemia and a raised ESR. Serum electrolytes and creatinine were within normal limits. X-ray of the spine was performed which revealed multiple bone lesions. The patient was counseled for the possibility of malignancy and referred to oncology for further workup. As the patient was a chronic smoker, chest computerized tomography (CT) was performed to screen for lung malignancy which showed no abnormalities. Furthermore, prostate-specific antigen, carcinoembryonic antigen, prostate examination, and abdominal imaging were performed to screen for prostate and colonic malignancy and were all unremarkable. Next up, multiple myeloma was suspected and further investigations including skeletal survey, serum calcium level, total protein/albumin ratio, serum and urine protein electrophoresis, and immunofixation studies were performed. Table 2 summarizes the serum electrolytes and other necessary results. Calcium was unusually within the normal range.

**Table 2:** Blood and urine workup PROT: Proteins; ALB: Albumin

Tests	Results	Reference Range
Serum calcium	10.26 mg/dl	8.1-10.4
Serum phosphorus	4.21 mg/dl	2.3-4.7
Beta-2-microglobulin	12,835 ng/ml	670-2134
Serum total PROT/ALB	2.58	1.2-2.1
Serum protein electrophoresis	No monoclonal gammopathy seen	-
Serum urine electrophoresis	No monoclonal gammopathy seen	-

We performed skeletal survey and immunofixation (Table 3). Skeletal survey revealed multiple lytic lesions in the skull, ribs, humerus, scapulae, and vertebrae while immunofixation showed elevated free light chain protein levels as seen in Table 3.

**Table 3:** Serum immunofixation Ig: Immunoglobulins

Test description	Observed value	Reference interval
Serum total proteins	7.70	6.40 to 8.20 g/dL
Serum Albumin	4.64	3.57 to 5.42 g/dL
Alpha 1 globulin	0.62	0.19 to 0.40 g/dL
Alpha 2 globulin	1.33	0.45 to 0.96 g/dL
Beta 1 globulin	0.42	0.30 to 0.59 g/dL
Beta 2 globulin	0.36	0.20 to 0.53 g/dL
Gamma globulin	0.33	0.71 to 1.54 g/dL
Albumin:Globulin ratio	1.51	1.1 to 2.2
“M” Band	Monoclonal Band not seen	Absent
IgA level, serum by nephelometry	11.80	70 to 400 mg/dL
IgG level, serum by nephelometry	319.00	700-1600 mg/dL
IgM serum by nephelometry	Below 4.24	40-230 mg/dL
Free Kappa (light chain)	46.30	3.3-19.4 mg/L
Free Lambda (light chain)	24.30	5.71-26.3 mg/L
Free Kappa/Lambda (light chain)	1.91	0.26-1.65 mg/L

**Figure 1:**
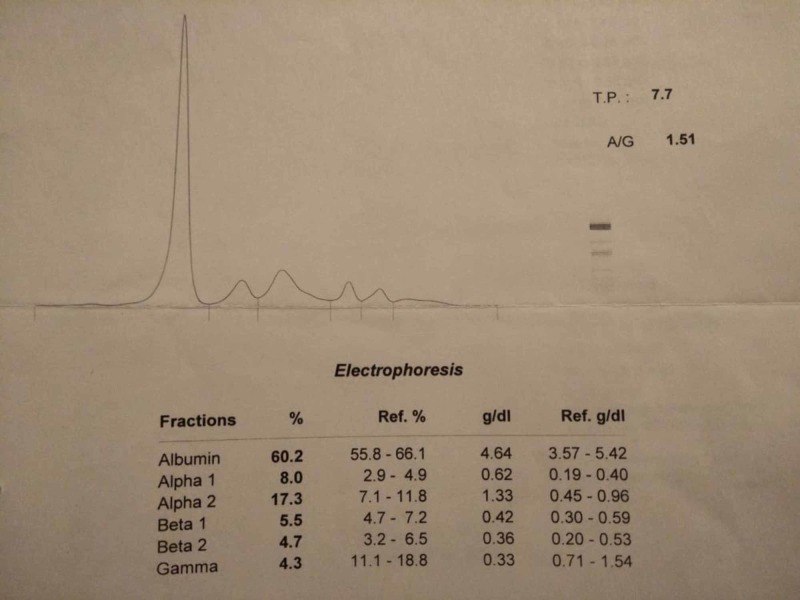
Serum protein electropheresis

We also did SPEP which is given below in figure 1. This case had a unique presentation of multiple myeloma as there was no evidence of monoclonal gammopathy (M protein spike) on SPEP however, elevated free light chain protein levels were found on immunofiaxation.

Further workup included urine Bence Jones protein and bone marrow biopsy. There was no trace of Bence Jones proteinuria. Bone marrow biopsy revealed a hypocellular bone marrow with plasma cell infiltrates of >10%, therefore the diagnosis of multiple myeloma was confirmed. Their results are shown in Table 4.

**Table 4:** Multiple myeloma workup

Test	Comments
Urine Bence Jones Protein	ABSENT
Bone marrow biopsy	>10% plasma cell infiltrates

**Figure 2:**
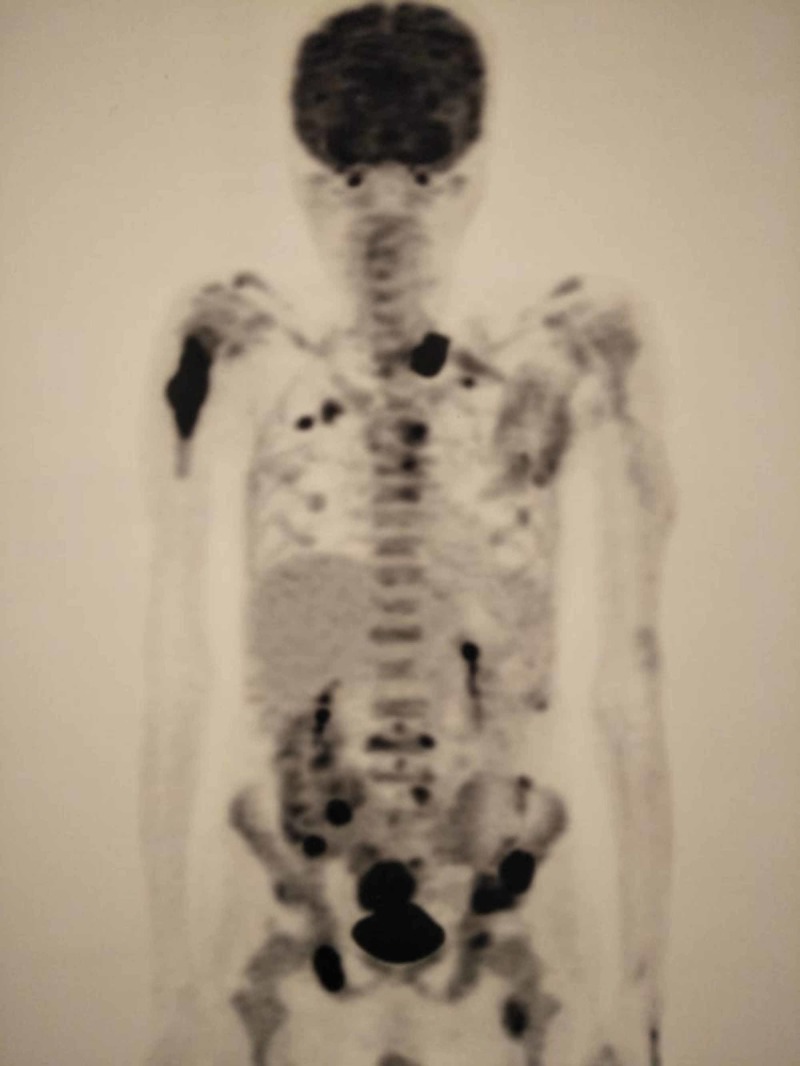
Positron emission tomography

We also did positron emission tomography (PET) scan which is shown in Figure 2. PET scan in the figure below shows multiple focuses of lytic lesions involving the right humerus, vertebrae, ribs, scapulae, and iliac crests. Prognosis considering stage three was explained to the relatives and he was started on bortenat, cyclophosphamide, and dexamethasone regimen (BOR-CY-DEX). On follow up, after completion of six cycles of BOR-CY-DEX regimen, he showed a remarkable improvement in general health and generalized bone pains were controlled by the use of fentanyl patch. His follow-up bone marrow biopsy revealed 1% of plasma cells.

## Discussion

Multiple myeloma is a consequence of abnormal plasma cell proliferation in the bone marrow and accounts for 10% of the hematological malignancies, hence it is uncommon. SPEP remains a gold standard test and is mostly used to detect the M protein spike in a myeloma patient. The confirmation of the diagnosis is aided by biochemical, radiological, and histological findings [4]. Rare sub-classification of multiple myeloma is a Non-Secreting Myeloma (NSM). The key diagnostic point that differentiates NSM from other subtypes of myeloma is the absence of monoclonal gammopathy on serum or urine protein electrophoresis or immunofixation. Protein electrophoresis is often the first test in the workup of myeloma and absence of M spike may lead to misdiagnosis of the NSM variant. Therefore, all the subtypes of multiple myeloma need to be comprehensively studied to aid in reaching an accurate diagnosis. NSM has been further divided into two variants, the “producer/ true” type, and the “non-producer” type. The producer type as the name suggests can synthesize immunoglobulin by the plasma cells but is unable to secrete it. The other non-producer type of NSM fails to produce the immunoglobulin at all [5]. Fewer cases of NSM will be reported if serum or urine is more accurately tested for traces of M protein. With the use of free light chain assays, many of the NSM based on SPEP or immunofixation results were found to have elevated free light chains in the serum, therefore further narrowing the diagnosis of NSM [6].

## Conclusions

In conclusion, a patient presenting with clinical features of multiple myeloma should undergo extensive workup involving CBC, serum chemistry, protein electrophoresis, immunofixation, free light chain studies, radiologic imaging, and bone marrow biopsy to conclude diagnosis of multiple myeloma with respect to their subtypes. The absence of monoclonal gammopathy on protein electrophoresis or normal immunofixation does not negate the diagnosis of multiple myeloma.
